# COVID-19 and Acute Sarcopenia

**DOI:** 10.14336/AD.2020.1014

**Published:** 2020-12-01

**Authors:** Carly Welch, Carolyn Greig, Tahir Masud, Daisy Wilson, Thomas A Jackson

**Affiliations:** ^1^Institute of Inflammation and Ageing, College of Medical and Dental Sciences, University of Birmingham, Birmingham, B15 2TT, UK.; ^2^MRC-Versus Arthritis Centre for Musculoskeletal Ageing Research, University of Birmingham and University of Nottingham, UK.; ^3^University Hospitals Birmingham NHS Trust, Birmingham, B15 2GW, UK.; ^4^School of Sport, Exercise, and Rehabilitation Sciences, University of Birmingham, Birmingham, B15 2TT, UK.; ^5^Birmingham Biomedical Research Centre, University of Birmingham and University Hospitals Birmingham NHS Foundation Trust, Birmingham, UK.; ^6^University of Nottingham, Nottingham, UK.; ^7^Nottingham University Hospitals NHS Trust, Nottingham, UK.

**Keywords:** COVID-19, sarcopenia, acute

## Abstract

The COVID-19 pandemic has had a devastating global impact, with older adults being most at risk of death from the disease. However, acute sarcopenia occurs in survivors of COVID-19; older adults and the most critically unwell patients are the most at risk. Acute sarcopenia is an under-recognised condition of acute muscle insufficiency, defined by declines in muscle function and/or quantity within six months, usually following a stressor event. This commentary reviews definition and mechanisms of acute sarcopenia in COVID-19 and suggests recommendations for research and clinical practice. Research should now focus on the longer-term consequences of acute sarcopenia in patients who have suffered from COVID-19. At the same time, clinicians need to be increasingly aware of the condition, and measurements of muscle strength, quantity, and physical performance should be embedded into clinical practice. Clinicians should consider the risks of acute sarcopenia when weighing up the risks and benefits of treatment (e.g. dexamethasone), and instigate multidisciplinary treatment including dietetics input.

Sarcopenia is a condition of extreme muscle insufficiency [1], defined by reduced muscle strength with reduced muscle quantity and/or muscle quality [2]. Severe sarcopenia is defined by additional demonstration of low physical performance. Cut-offs are taken as more than two standard deviations below the mean of a young healthy reference population [2]. Sarcopenia is associated with limitations upon physical function and quality of life [3], increased risk of falls [4], and increased mortality [5]. It has been considered a precursor or physical manifestation of frailty, a state of increased vulnerability [6]. Acute sarcopenia is defined by the European Working Group on Sarcopenia in Older People 2 (EWGSOP2) as incident sarcopenia within six months, normally following a stressor event [2]. Acute sarcopenia most commonly occurs in hospitalised patients. Older adults with frailty are considered to be most vulnerable [7], however, it is increasingly recognised that sarcopenia can develop at any age [8]. Previously robust individuals may develop acute sarcopenia following severe illness and admission to critical care [7]. Acute sarcopenia is akin to acute organ insufficiency elsewhere e.g. Acute Kidney Injury [7].

Sarcopenia is common in hospitalised populations [9-12] and associated with increased risk of adverse outcomes; low muscle quantity has been associated with increased risk of post-operative infections and mortality, as well as increased rehabilitation needs and length of stay in surgical populations [13-16]. Sarcopenia has been associated with reduced diaphragmatic muscle thickness [17]. Acute declines in diaphragmatic muscle thickness in hospitalised patients can provoke respiratory failure, and necessitate prolonged mechanical ventilation in critically unwell patients [18]. Although the current definition of sarcopenia refers to an extreme level of muscle insufficiency, it is also recognised that relative changes for individuals that do not meet the criteria for sarcopenia may be themselves individually significant [7]. The longer-term effects of acute sarcopenia remain unknown [7]. However, acute sarcopenia will lead to significant detriments in physical function, at least in the short-term, and it has been hypothesised that periods of acute sarcopenia may increase the risk of developing chronic sarcopenia [7].

Muscle loss in acute sarcopenia is due to an imbalance in muscle homeostasis with increased muscle degradation and reduced muscle synthesis. Muscle degradation is a term which broadly describes the loss of muscle via both reduction in muscle fibre size (atrophy) and reduction in the number of muscle fibres (hypoplasia). Hypoplasia is believed to occur secondary to motor neurone death and muscle fibre denervation and atrophy secondary to proteolytic pathways [19]. Older adults exhibit a blunted synthetic response to both feeding and exercise, termed anabolic resistance, compared to young adults [20-22]. However, in acute illness catabolic pathways are likely to be more relevant.

Unfortunately, measurement of muscle strength or muscle quantity are not currently fully integrated into routine clinical care, although anthropometry may be performed as part of a dietetics assessment [23]. Therefore, acute sarcopenia is an often-unconsidered organ insufficiency, as it requires a comprehensive evaluation of the patient to be identified; it cannot be identified through remote review of biochemistry or vital signs alone. Without consideration of dynamic changes in muscle strength and quantity, acute sarcopenia may go unnoticed until it is clinically extreme. The importance of muscle insufficiency may be disregarded by clinicians when the initial focus of care is on survival. However, it is always important to consider the wishes of the patient themselves, since for many, survival with good physical function may be just as important, if not more important than survival itself [24]. Additionally, deprioritising the muscle within early clinical care risks further challenges as rehabilitation is likely to be prolonged and more difficult [25], leading to increased lengths of stay, and increased vulnerability to further illnesses, and a negative spiralling effect.

**Figure 1.**
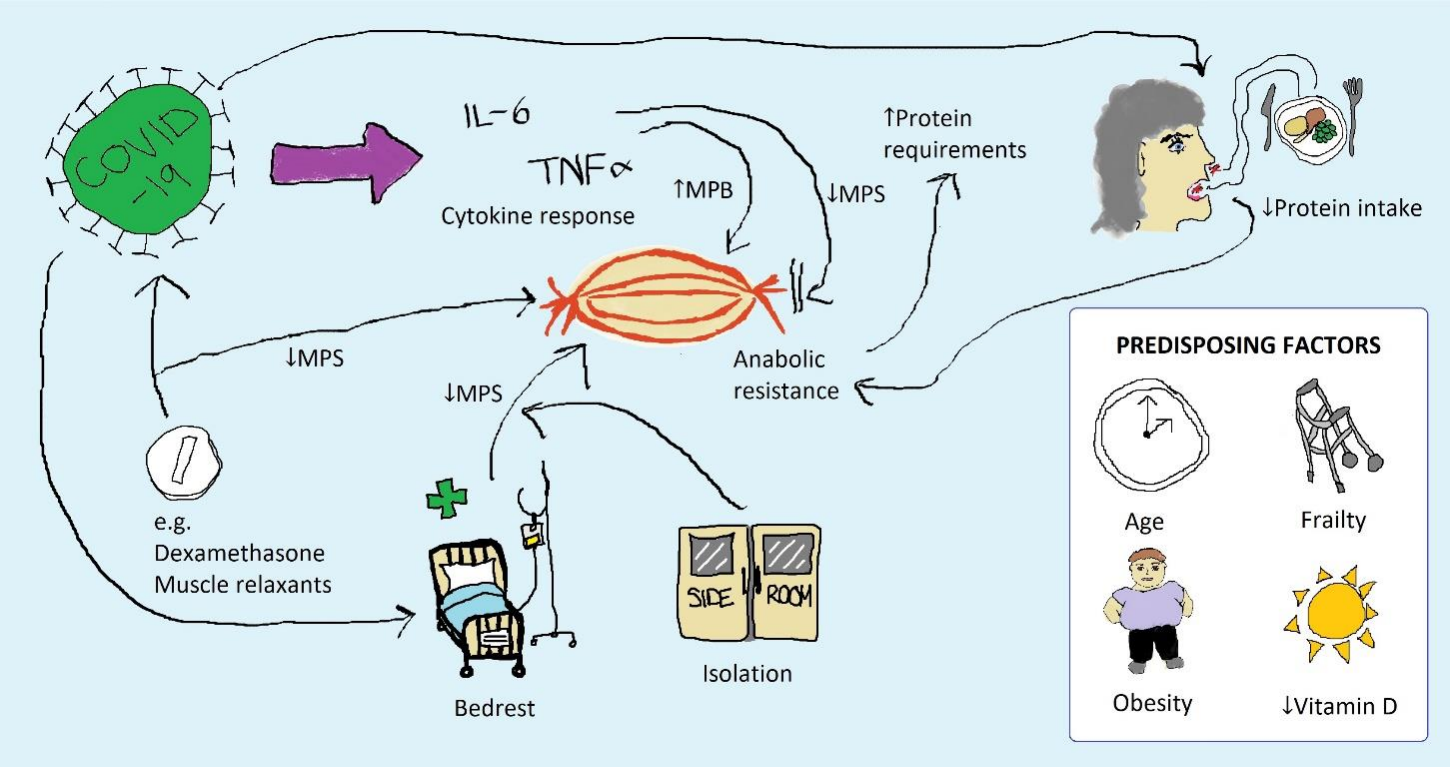
Mechanisms of acute sarcopenia development with COVID-19. Precipitating factors for acute sarcopenia with COVID-19 are demonstrated by pathways and predisposing factors are shown separately. *MPB = Muscle Protein Breakdown; MPS = Muscle Protein Synthesis*

## Mechanisms of acute sarcopenia with COVID-19

Coronavirus disease 2019 (COVID-19) is a severe acute infectious disease caused by the Severe Acute Respiratory Syndrome Coronavirus 2 (SARS-CoV-2) [26]. The global pandemic caused by this virus has resulted in unprecedented global healthcare demand. The initial focus of care within the pandemic was to prevent unnecessary deaths from the infection [27]; older adults are known to be particularly vulnerable to the effects of the illness, with age associated with increased mortality [28]. However, it has now become clear that survivors of COVID-19 are at increased risk of acute sarcopenia [29, 30], with worsening muscle insufficiency identified across a multitude of settings [31, 32]. Figure 1 demonstrates proposed mechanisms of acute sarcopenia with COVID-19 as described below.

### Inflammation

COVID-19 is known to be associated with significant systemic inflammation and a subset of patients will experience a severe cytokine response. Serum concentrations of inflammatory cytokines including Tumour Necrosis Factor Alpha (TNF-α) have been shown to be higher in patients with COVID-19 requiring critical care treatment [33]. This has negative consequences upon muscle protein synthesis; TNF-α decreases messenger Ribonucleic Acid (mRNA) translational efficiency through alterations in Eukaryotic translation initiation factor 4E (eIF-4E) availability [34]. This results in a state of anabolic resistance, which necessitates a requirement for higher protein intake to stimulate muscle protein synthesis. Rodent models of sepsis have also shown increases in the muscle-specific ubiquitin ligases (Muscle Ring Finger 1, MuRF-1 and Muscle Atrophy F-box, MAFbx) in relation to inflammation [35]. Ageing itself is associated with increased cellular senescence. Although senescent cells represent a state of arrested cell growth, they also secrete high levels of inflammatory cytokines [36]. Thus, the effects of inflammation and acute illness may be exacerbated with age.

### Vitamin D

Vitamin D deficiency has been implicated in sarcopenia; muscle biopsies from individuals with vitamin D deficiency have shown atrophy of Type II muscle fibres [37]. Vitamin D has also been hypothesised to affect the immune response to respiratory infections [38]. However, vitamin D deficiency is increasingly recognised to occur as a consequence of inflammation rather than a cause [39]. Thus, vitamin D deficiency in critical illness may simply represent a biomarker of heightened inflammation. Some studies have demonstrated an association between low vitamin D and development of COVID-19 [40, 41], but no causal relationship has been demonstrated after adjusting for multiple confounders [40].

### Obesity

Obesity has been demonstrated as an adverse prognostic factor in COVID-19, being associated with increased risk of hospitalisation, critical care admission, and mortality [42]. Obesity in itself is associated with increased systemic inflammation, which may exacerbate the effects of acute illness upon muscle metabolism. Sarcopenic obesity is a recognised condition defined by reduced muscle mass with increased fat mass. Sarcopenic obesity may also be associated with ectopic deposition of fat and intramyocellular lipid deposition, thus affecting the quality of muscle [43]. This effect may have predisposed individuals with obesity and catabolic states from their illness to significant declines in muscle function in association with declines in muscle quantity.

### Critical care admission

The most marked individual declines in muscle function in hospitalised patients with COVID-19 were seen in patients who required admission to critical care [31], relating to marked elevations in systemic inflammation, prolonged bedrest, and use of muscle relaxants to aid prone positioning and reduce risk of viral spread [44]. Acute sarcopenia has been demonstrated in patients who were previously fit and active prior to hospital admission, but experienced severe declines in their muscle function. In many patients, this also led to a state of induced frailty [45], with increased vulnerability to stressor events [46]. Induced frailty in itself led to further compromises to the immune system, making the patients more vulnerable during their recovery [45].

### Nutrition

As described, research suggests that heightened inflammation in COVID-19 is associated with catabolic states and anabolic resistance, leading to increased nutritional demand, particularly protein. Despite this, many patients with COVID-19 struggled to meet even basic requirements. Loss of sense of taste or smell are recognised symptoms of COVID-19, that may occur in up to two thirds of cases [47]. This leads to diminished appetite. In addition, upregulation of proinflammatory cytokines, as seen in COVID-19, is associated with induction of leptin and anorexia [48]. Combined with the effects of anorexia of ageing [49], older adults may be particularly vulnerable to these effects. In addition, sarcopenia in itself has been shown to be associated with weakness in masticatory muscles, which may further exacerbate diminished food intake [50].

### Bedrest and physical activity

Importantly, many patients hospitalised with COVID-19 suffered prolonged periods of bedrest and reduced physical activity. Depending on hospital models, this may have been further exacerbated by isolation policies preventing mobilisation outside of smaller ward areas or side rooms. Even in those who did not require hospitalisation, there is evidence that patients who contracted COVID-19 suffered from immense fatigue. This is likely to have reduced their physical activity [29]. During the COVID-19 pandemic, most countries also placed restrictions on activities that could be conducted in order to combat spread of the virus. Older adults were considered the most vulnerable, and often had the greatest restrictions placed on them. This is likely to have significantly affected the amount of physical activity engagement by older adults during this time period [51], making them increasingly vulnerable if they did require hospitalisation. Bedrest has been shown to be associated with declines in muscle quantity, strength, and aerobic performance in healthy volunteer studies [52]. This effect is exacerbated by age [53]. Bedrest leads to reduced muscle protein synthesis via altered expression of ubiquitin ligases in healthy young adults (MuRF-1 and MAFbx) [54, 55].

### COVID-19 treatment

The effect of treatment initiated for COVID-19 should also be considered. The Randomised Evaluation of COVID-19 Therapy (RECOVERY) trial demonstrated a survival benefit with dexamethasone, particularly when given to the most unwell patients [56]. However, medically-induced hypercortisolaemia has been shown to induce muscle loss with bedrest compared to bedrest alone [57]. Dexamethasone itself has been shown to up-regulate MuRF-1 and MAFbx 10-fold in rodent models [58]. Therefore, dexamethasone may increase risk of acute sarcopenia in already vulnerable patients.

### Recovery

Previous research completed in patients with Acute Respiratory Distress Syndrome has demonstrated a decrease in lean muscle mass during the year following discharge from critical care [59]. This suggests a lasting effect preventing the synthesis of new muscle following acute severe illness. Murine models of sepsis have demonstrated that muscle regeneration capabilities following sepsis are severely limited; increased fibrosis as well as a decreased number and function of satellite cells [60]. It is hypothesised that prolonged immune changes as a consequence of sepsis or COVID-19 result in a reduction in the body’s ability to synthesise muscle with protracted sequalae of acute sarcopenia.

## Recommendations for research and clinical practice

Research that aims to characterise changes in muscle quantity, quality, and function in hospitalised patients with COVID-19 and other conditions [61] is imperative towards increasing our understanding of this condition. Mechanistic and biomarker-driven studies will enable increased understanding of the underlying molecular pathways to guide risk stratification in patients, as well as assisting with development of novel treatment pathways. Longer term cohort studies, such as the Post-Hospitalisation COVID-19 (PHOSP-COVID) study [62], which includes a sarcopenia subgroup, will enable detailed phenotyping of patients who have developed acute sarcopenia, and increased understanding of recovery within one year of illness. Where feasible, muscle biopsies may provide valuable mechanistic characterisation during the acute phase of illness and recovery; these are planned as part of the PHOSP-COVID study [62]. Although it is recognised that muscle biopsies are often less feasible in older people with frailty [63]. Targeted interventional studies will also enable identification of cost-effective strategies to prevent and treat acute sarcopenia in further patients who are hospitalised as the pandemic continues, with the potential for use in other illnesses.

At the same time, clinicians need to be increasingly aware of the problem of acute sarcopenia. Where possible, clinicians should integrate serial measurements of muscle strength, physical performance, and muscle quantity into their clinical practice, enabling them to identify early when there is a change, and assess responsiveness to treatment and therapy. Without any form of monitoring, acute sarcopenia will not be identified until clinically extreme. Muscle strength can be measured using a dynamometer, where available; assessment of chair stands is an alternative where a dynamometer is not available and the patient is able to sit in a chair [2]. Ultrasound and bioelectrical impedance analysis are options for measurement of muscle quantity [2], and even anthropometry (e.g. calf size) [64] can be of value in a time-limited environment, when no other methods are available. Importantly, these methods can be implemented even when it is not possible to assess muscle function (e.g. intubated patients in critical care). In awake patients, the Hierarchical Assessment of Balance and Mobility (HABAM) is an instrument that provides dynamic assessment of in-bed balance, transfers, and ambulation [65]; integrating this into clinical practice can allow monitoring of in-hospital function in the same way as vital signs.

In any care model for patients suffering from or recovering from COVID-19, treatment needs to enlist a multidisciplinary approach [25]. Early dietetics involvement is paramount to optimise protein intake particularly. In some cases, up to 2g protein/kg/day may be required [66], and this may even necessitate a period of nasogastric feeding [67]. An enhanced physiotherapy programme should focus on graded response, assessing what is feasible within the clinical environment. Whilst infection control policies will necessitate isolation from patients without COVID-19, clinical areas should enable mobilisation as much as possible e.g. by cohorting patients with COVID-19 together. Specialised wards with strength training equipment will help to build a supportive environment focused around rehabilitation. However, ideally a rehabilitation ethos and therapy provision should be embedded throughout the hospital, to enable treatment to commence even in the early acute phase. Clinicians should carefully consider the risks and benefits of all treatment. Dexamethasone is now given routinely to hospitalised patients with COVID-19 requiring oxygen or critical care admission. However, clinicians should be aware of the potential for increased risk of muscle loss if prolonged courses are given. The longer-term consequences of COVID-19 and acute sarcopenia are unknown. We recommend incorporation of muscle function evaluation in services providing clinical follow-up to enable referral to rehabilitation, dietetics, and other services as applicable.

## Conclusions

Acute sarcopenia is a condition of severe acute muscle insufficiency that normally occurs following a stressor event; COVID-19 is a particularly aggressive insult. Further research is needed to help characterise this condition and understand the longer-term consequences. This will enable the development of targeted interventions. Meanwhile, clinicians should be aware of this condition within their own practice and seek early multidisciplinary team involvement.
